# Delirium in hip fracture patients admitted from home during the COVID-19 pandemic is associated with higher mortality, longer total length of stay, need for post-acute inpatient rehabilitation, and readmission to acute services: the IMPACT Delirium study

**DOI:** 10.1302/2633-1462.46.BJO-2023-0045.R1

**Published:** 2023-06-16

**Authors:** Rose S. Penfold, Andrew J. Hall, Atul Anand, Nick D. Clement, Andrew D. Duckworth, Alasdair M. J. MacLullich

**Affiliations:** 1 Edinburgh Delirium Research Group, Ageing and Health, Usher Institute, University of Edinburgh, Edinburgh, UK; 2 Scottish Hip Fracture Audit, Edinburgh, UK; 3 Department of Orthopaedics, Golden Jubilee University National Hospital, Clydebank, UK; 4 Edinburgh Orthopaedics, Royal Infirmary of Edinburgh, Edinburgh, UK; 5 Centre for Cardiovascular Science, University of Edinburgh, Edinburgh, UK; 6 NHS Lothian, Edinburgh, UK; 7 Department of Orthopaedics & Usher Institute, University of Edinburgh, Edinburgh, UK

**Keywords:** Delirium, Hip fracture, Geriatrics, Perioperative medicine, Clinical audit, inpatient rehabilitation, COVID-19 infection, Anesthesiologists, prognosis, trauma, logistic regression, acute hip fracture, frailty

## Abstract

**Aims:**

Delirium is associated with adverse outcomes following hip fracture, but the prevalence and significance of delirium for the prognosis and ongoing rehabilitation needs of patients admitted from home is less well studied. Here, we analyzed relationships between delirium in patients admitted from home with 1) mortality; 2) total length of hospital stay; 3) need for post-acute inpatient rehabilitation; and 4) hospital readmission within 180 days.

**Methods:**

This observational study used routine clinical data in a consecutive sample of hip fracture patients aged ≥ 50 years admitted to a single large trauma centre during the COVID-19 pandemic between 1 March 2020 and 30 November 2021. Delirium was prospectively assessed as part of routine care by the 4 A’s Test (4AT), with most assessments performed in the emergency department. Associations were determined using logistic regression adjusted for age, sex, Scottish Index of Multiple Deprivation quintile, COVID-19 infection within 30 days, and American Society of Anesthesiologists grade.

**Results:**

A total of 1,821 patients were admitted, with 1,383 (mean age 79.5 years; 72.1% female) directly from home. Overall, 87 patients (4.8%) were excluded due to missing 4AT scores. Delirium prevalence in the whole cohort was 26.5% (460/1,734): 14.1% (189/1,340) in the subgroup of patients admitted from home, and 68.8% (271/394) in the remaining patients (comprising care home residents and inpatients when fracture occurred). In patients admitted from home, delirium was associated with a 20-day longer total length of stay (p < 0.001). In multivariable analyses, delirium was associated with higher mortality at 180 days (odds ratio (OR) 1.69 (95% confidence interval (CI) 1.13 to 2.54); p = 0.013), requirement for post-acute inpatient rehabilitation (OR 2.80 (95% CI 1.97 to 3.96); p < 0.001), and readmission to hospital within 180 days (OR 1.79 (95% CI 1.02 to 3.15); p = 0.041).

**Conclusion:**

Delirium affects one in seven patients with a hip fracture admitted directly from home, and is associated with adverse outcomes in these patients. Delirium assessment and effective management should be a mandatory part of standard hip fracture care.

Cite this article: *Bone Jt Open* 2023;4(6):447–456.

## Introduction

Delirium is an acute-onset, fluctuating neuropsychiatric syndrome characterized by disturbed consciousness, attention, and cognition. It is common in patients with an acute hip fracture, and is associated with adverse outcomes, including reduced mobility, prolonged hospital stay, in-hospital mortality, and higher care requirements following discharge.^1^ There is increasing recognition of the importance of delirium in the management and prognosis following hip fracture. Local and national hip fracture guidelines now urge effective delirium detection and care.^2-4^

The prevalence of delirium in hip fracture patients has mainly been investigated in research studies, with delirium assessment performed by research or specialist clinical teams. Most studies have assessed postoperative delirium (POD) rates: in a 2015 systematic review of ten studies, the prevalence of POD ranged from 13% to 56%.^5^ Three recent studies reported POD prevalences of 19%, 22%, and 25%.^1,6,7^ A smaller group of studies have reported preoperative delirium prevalences, of 19% (only home-dwelling patients included), 58%, and 18%.^8-10^

Another source of data on delirium in hip fracture is large clinical registries. However, according to the most recent published reports, delirium is prospectively assessed as part of routine care in a minority: the National Hip Fracture Database (NHFD; England, Wales and Northern Ireland), the Scottish Hip Fracture Audit (SHFA), the Irish Hip Fracture Database, and the Australian and New Zealand Hip Fracture Registry.^11-15^ Of these, only the NHFD has reported POD prevalence rates in three subsets of data: 25% in a single-centre study of 1,224 patients (2016 to 2018), 26% in a single centre of 175 patients (2018 to 2019), and 29% in 107,028 patients from the whole NHFD (2018 to 2019).^16-18^ In a USA National Surgical Quality Improvement Program (NSQIP) study, in which delirium was ascertained retrospectively using International Classification of Diseases (ICD)-9 codes, the POD rate in hip fracture patients was 27%.^19^

Several studies have assessed outcomes following delirium in hip fracture patients.^1,19,20^ These have analyzed whole cohorts, comprising patients admitted from home, all care settings, and those sustaining fractures as inpatients. One study using local NHFD data reported that POD was associated with increased mortality at 30 days and one year.^17^ Less is known about delirium in patients admitted from home, including the prevalence and significance of delirium for prognosis and ongoing rehabilitation needs in these patients. Returning to live at home on discharge is important, with days alive and at home following a hip fracture validated as a patient-centred outcome for patients aged > 50 years.^21^ In a study of 207 patients admitted directly from private homes, Krogseth et al^8^ reported an inpatient delirium incidence of 39%, and associations with higher mortality and risk of new institutionalization. Determining whether similar associations exist in large, population-representative datasets with routine delirium assessment is important for clinical practice.

In Scotland, hip fracture services are delivered in accordance with the Scottish Standards of Care for Hip Fracture Patients (SSCHFP). Delirium assessment is mandated for all patients on admission using the 4 A’s Test (4AT).^22^ This study aim was to determine any associations of delirium in patients admitted from home with: 1) mortality at 30 and 180 days; 2) total length of stay in acute and subacute care settings; 3) need for post-acute inpatient rehabilitation; and 4) readmission to hospital from home within 180 days.

## Methods

This study is reported in line with STROBE guidelines for observational studies.

### Study population

This cohort study was based on the International Multicentre Project Auditing COVID-19 in Trauma & Orthopaedics (IMPACT) Hip Fracture Audit,^23,24^ using prospectively collected, retrospectively validated clinical audit data from a single high-volume orthopaedic major trauma centre in Scotland. IMPACT is a global multicentre project, originally purposed to coordinate urgent collaborative research, report near real-time data, and guide the clinical response to COVID-19. All patients ≥ 50 years admitted with an acute hip fracture between 1 March 2020 and 30 November 2021 were included. Direct admission from home was determined based on contemporaneous clinical documentation, coded by specialist clinical auditors, and validated using live electronic health records (EHRs) by a study author (AJH). Patients were included if they had an intracapsular or extracapsular fracture of the proximal femur, up to and including the subtrochanteric region. Fractures around an existing implant and isolated fractures of the public ramus, acetabulum, or greater trochanter were excluded, since these patients were excluded from data collection for national audit purposes at the time of the study.

### Data collection

Data were collected from EHRs (TrakCare; InterSystems Corporation, USA) and clinical documentation by trained specialist audit coordinators as part of routine audit activity. Data were collated by specialist auditors, and verified by a senior analyst independent of the study. Variables included: patient demographics (age, sex, type of residence on admission); Scottish Index of Multiple Deprivation (SIMD); patient factors (American Society of Anesthesiologists (ASA) grade); COVID-19 within 30 days; injury factors (fracture type); inpatient care factors (type of operation; length of stay in acute and subacute inpatient settings); delirium assessed using the 4AT; discharge destination; number and date of readmissions; and mortality status up to 180 days. SIMD is an area-based relative measure of deprivation.^25^ COVID-19 infection was considered within 30 days, as this was previously demonstrated to be independently associated with increased mortality.^23^ Delirium was defined as a 4AT score ≥ 4. The 4AT is a well-validated, brief clinical tool for delirium recommended in numerous guidelines and hip fracture registries.^12,13,26,27^ The first available 4AT score from the emergency department (ED) was used; if none recorded, then the first 4AT score from the admission orthopaedic ward was used instead. The vast majority of 4AT assessments were performed within 24 hours of admission, as per admission protocol. Data underwent verification through manual cross-referencing against live EHRs by AJH, and additional variables were collected manually including mortality status following admission, and date of death up to 180 days. The EHRs for all patients were reviewed for a minimum of 180 days after date of admission, or until date of death.

Patients with no 4AT score from the ED or admission orthopaedic ward were excluded. Patients from outside the service catchment area were included in all analyses except readmission, where they were excluded due to lack of reliable follow-up. Data were compiled using the bespoke IMPACT Audits data collection tool using data-validated fields to ensure coding accuracy and consistency.^28^ This dataset has been used in previously published studies.^29,30^ Data were collected and handled as part of service evaluation in accordance with UK Caldicott principles.

### Statistical analysis

Statistical analyses were performed using RStudio version 4.2.1 and packages: finalfit; dplyr; survival; survminer; TableOne, epiDisplay, and tidyverse (Integrated Development for R; Rstudio, PBC, USA). Delirium was coded as a binary variable: 4AT score ≥ 4 suggestive of delirium.^22,26^ Continuous variables were assessed for differences between groups using an independent-samples *t*-test (parametric) or Mann-Whitney U test (non-parametric), and categorical variables using a chi-squared test. Multivariable logistic regression was used to analyse associations of delirium with: 1) mortality at 30 and 180 days; 2) need for post-acute inpatient rehabilitation; and 3) for patients discharged from the acute stay to a home within the catchment area, likelihood of readmission to an acute hospital within 180 days. All multivariable models included covariates: age, sex, SIMD quintile, COVID-19 within 30 days, and ASA grade. A p-value of < 0.05 was deemed significant. Assumptions of logistic regression were tested and satisfied.

### Supplementary analyses

Supplementary analyses were performed to assess baseline characteristics and outcomes in patients admitted from higher care settings, including care homes, rehabilitation facilities, and transfer from other acute care settings. A further multivariable logistic regression analysis was performed to analyse associations of delirium with mortality at 30 and 180 days in patients admitted from all care settings. This included the same covariates as the above regression models (age, sex, SIMD quintile, COVID-19 within 30 days, ASA grade), with an additional binary variable for type of residence on admission (home/higher care setting). A p-value of < 0.05 was deemed significant.

### Sensitivity analyses

Sensitivity analyses were performed including only patients with a 4AT score from the ED. In these patients it is known that any delirium was present on admission, and that assessment was preoperative.

## Results

### Delirium prevalence

**Fig. 1:**
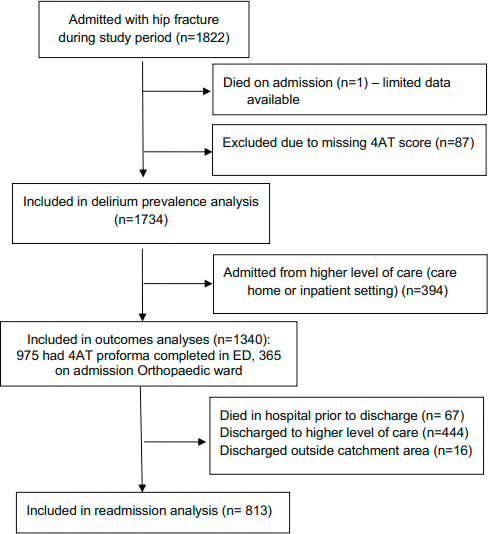
Flow chart demonstrating inclusion and exclusion of patients in the study. ED, emergency department.

The study flowchart is shown in Figure 1. A total of 1,822 patients (mean age 80.7 years; 71.7% female) were admitted during the study period. One died within hours of admission and was excluded. A total of 87 (4.8%) had missing 4AT scores, and were excluded. Of the 1,734 included patients, 460 (26.5%) had delirium.

There were 1,383 (mean age 79.5 years; 72.1% female) patients admitted directly from home. Of these, 43 had missing 4AT scores and were excluded. Of the 1,340 included patients (mean age 79.5 years; 72.2% female), 189 (14.1%) had delirium. In the remaining 394 patients admitted from care homes (283 (71.8%)), rehabilitation facilities (31 (7.9%)), and transferred from inpatient settings (80 (20.3%)), delirium prevalence was 68.8%.

### Baseline characteristics

Demographics of the 1,340 patients admitted from home are shown in Table I, stratified by delirium status. Patients with delirium were older and had a higher ASA grade, indicating greater severity of comorbid disease. There was no difference in the prevalence of COVID-19 within 30 days between the groups.

**Table I.** Patient baseline characteristics according to delirium status ascertained on admission for patients admitted from home. Univariable analyses were used to assess the difference between those with and without delirium for each variable. Delirium = 4 AT ≥ 4.

Variable	Whole cohort (n = 1,340)	Delirium (n = 189)	No delirium (n = 1,151)	p-value
Mean age, yrs (SD)	79.5 (10.3)	82.9 (8.9)	79.0 (10.4)	< 0.001*
**Age (yrs), n**				< 0.001†
50 to 59	69	7	62	
60 to 69	154	7	147	
70 to 79	356	36	320	
80 to 89	553	98	455	
90+	208	41	167	
Female sex, n (%)	968 (72.2)	129 (68.3)	839 (72.9)	0.218†
**SIMD quintile, n (%)**				0.078†
1 (most deprived)	168 (12.6)	15 (7.9)	153 (13.4)	
2	297 (22.3)	52 (27.5)	245 (21.4)	
3	221 (16.6)	31 (16.4)	190 (16.6)	
4	236 (17.7)	39 (20.6)	197 (17.2)	
5 (least deprived)	412 (30.9)	52 (27.5)	360 (31.4)	
**ASA grade, n (%)**				< 0.001†
1 (healthy)	38 (2.8)	1 (0.5)	37 (3.2)	
2	413 (30.8)	22 (11.6)	391 (34.0)	
3	789 (58.9)	136 (72.0)	653 (56.7)	
4	88 (6.6)	23 (12.2)	65 (5.6)	
5 (moribund)	12 (0.9)	7 (3.7)	5 (0.4)	
COVID-19 positive (< 30 days), n (%)	53 (4.0)	10 (5.3)	43 (3.7)	0.415†

* Independent-samples *t*-test

† Chi-squared test

ASA, American Society of Anesthesiologists; SD, standard deviation; SIMD, Scottish Index of Multiple Deprivation.

### Univariable analyses

Outcomes of the 1,340 patients admitted from home are shown in Table II, stratified by delirium status. Patients with delirium had a higher mortality rate at 30 and 180 days. Median total length of stay was 20 days longer for patients with delirium, versus those without (p < 0.001, Mann-Whitney U test). Overall, 444 patients (33.1%) went to post-acute inpatient rehabilitation facilities following the acute hospital stay (37 to care homes, 363 to rehabilitation facilities, and 44 to other acute care settings). Only 62/189 (32.8%) patients with delirium directly returned to private homes following the acute stay, versus 767/1151 (66.6%) of those without delirium (p < 0.001, chi-squared test).

**Table II.** Patient outcomes according to delirium status, as assessed on admission for patients admitted from home. Univariable analyses assess the difference between those with and without delirium for each variable. Delirium = 4AT ≥ 4.

Variable	Whole cohort (n = 1,340)	Delirium (n = 189)	No delirium (n = 1,151)	p-value
**Mortality, n (%)**				
Within 30 days	63 (4.7)	21 (11.1)	42 (3.6)	< 0.001*
Within 180 days	197 (14.7)	54 (28.6)	143 (12.4)	< 0.001*
**Discharge destination following acute hospital stay, n (%)**				< 0.001*
Own home	829 (61.9)	62 (32.8)	767 (66.6)	
Care home	37 (2.8)	10 (5.3)	28 (2.3)	
Rehabilitation facility	363 (27.1)	82 (43.4)	281 (24.4)	
Other acute care	44 (3.3)	9 (4.8)	35 (3.0)	
Died in hospital	67 (5.0)	26 (13.8)	41 (3.6)	
Discharge to post-acute rehabilitation, n (%)	444 (33.1)	101 (53.4)	343 (29.8)	< 0.001*
**Median total length of stay in inpatient facilities, days (IQR)**	16 (9 to 36)	34 (16 to 66)	14 (8 to 31)	< 0.001†
	**Patients discharged home in catchment area (n = 813)**	**Delirium (n = 61)**	**No delirium (n = 752)**	
**Readmission, n (%)**				
Within 30 days	153 (18.8)	22 (36.1)	131 (17.4)	0.001*
Within 180 days	307 (37.8)	36 (59.0)	271 (36.0)	0.001*

* Chi-squared test.

† Mann-Whitney U test.

IQR, interquartile range.

### Multivariable analyses

The following results relate to those patients admitted directly from home. In multivariable analyses adjusting for age, sex, SIMD, ASA grade, and COVID-19 within 30 days, delirium was independently associated with a higher 180-day mortality rate (odds ratio (OR) 1.69 (95% confidence interval (CI) 1.13 to 2.54); p = 0.011) (Table III). Delirium was independently associated with increased likelihood of requiring post-acute inpatient rehabilitation following the acute stay (OR 2.80 (95% CI 1.97 to 3.96); p < 0.001) (Table IV). In total, 16 patients were discharged to residences outside the catchment area and were excluded from readmission analysis. In the 813 patients who went home within the catchment area, delirium was independently associated with an increased risk of readmission to an acute hospital within 180 days (OR 1.79 (95% CI 1.02 to 3.15); p = 0.041) (Table V).

**Table III.** Uni- and multivariable logistic regression analysis of factors associated with 30- and 180-day mortality for patients admitted from home. Multivariable analysis was adjusted for age, sex, Scottish Index of Multiple Deprivation, American Society of Anesthesiologists grade, and COVID-19 within 30 days.

Variable	Within 30 days, OR (95% CI)		p-value	Within 180 days, OR (95% CI)		p-value
	Univariable analysis	Multivariable analysis		Univariable analysis	Multivariable analysis	
**Age**						
Per additional year	1.03 (1.00 to 1.06)	1.04 (1.00 to 1.07)	0.037	1.05 (1.03 to 1.07)	1.05 (1.03 to 1.07)	< 0.001
**Sex**						
Male	Reference	Reference		Reference	Reference	
Female	0.70 (0.41 to 1.19)	0.63 (0.34 to 1.14)	0.126	0.55 (0.40 to 0.76)	0.49 (0.34 to 0.69)	< 0.001
**SIMD quintile**						
1 (most deprived)	Reference	Reference		Reference	Reference	
2	0.72 (0.31 to 1.69)	0.61 (0.24 to 1.53)	0.293	1.08 (0.63 to 1.82)	0.95 (0.54 to 1.67)	0.854
3	0.75 (0.30 to 1.84)	0.47 (0.16 to 1.39)	0.173	0.54 (0.29 to 1.02)	0.42 (0.21 to 0.84)	0.014
4	0.63 (0.25 to 1.58)	0.50 (0.18 to 1.39)	0.187	0.93 (0.53 to 1.63)	0.74 (0.40 to 1.36)	0.333
5 (least)	0.85 (0.39 to 1.84)	0.65 (0.27 to 1.55)	0.331	1.23 (0.75 to 2.01)	1.04 (0.61 to 1.79)	0.874
**ASA grade** *						
1 (healthy)	-	-	-	-	-	-
2	0.27 (0.10 to 0.68)	0.32 (0.12 to 0.84)	0.021	0.32 (0.20 to 0.50)	0.41 (0.25 to 0.65)	< 0.001
3	Reference	Reference		Reference	Reference	
4	3.07 (1.50 to 6.29)	2.80 (1.33 to 5.88)	0.007	3.09 (1.93 to 4.95)	3.23 (1.97 to 5.29)	< 0.001
5 (moribund)	-	-	-	-	-	-
**Delirium status:** 4AT ≥ 4	3.28 (1.90 to 5.68)	1.72 (0.89 to 3.34)	0.109	2.80 (1.95 to 4.02)	1.69 (1.13 to 2.54)	0.011
**COVID-19-positive (< 30 days)**	2.74 (1.13 to 6.68)	3.02 (1.17 to 7.78)	0.022	1.54 (0.78 to 3.05)	1.41 (0.67 to 2.96)	0.359

* No patients ASA grade 1 died within 180 days; all patients ASA grade 5 died within 30 days.

ASA, American Society of Anesthesiologists; CI, confidence interval; OR, odds ratio; SIMD, Scottish Index of Multiple Deprivation.

**Table IV.** Uni- and multivariable logistic regression analysis of factors associated with requirement for post-acute inpatient rehabilitation following the acute inpatient stay (including care home, rehabilitation facility, or other acute care setting) for patients admitted from home. Multivariable analysis adjusted for age, sex, Scottish Index of Multiple Deprivation, American Society of Anesthesiologists grade, and COVID-19 within 30 days.

Variable	OR (95% CI)		p-value
	Univariable analysis	Multivariable analysis	
**Age**			
Per year	1.06 (1.05 to 1.07)	1.05 (1.04 to 1.06)	< 0.001
**Sex**			
Male	Reference	Reference	
Female	0.97 (0.75 to 1.24)	0.92 (0.70 to 1.21)	0.537
**SIMD quintile**			
1 (most deprived)	Reference	Reference	
2	1.24 (0.84 to 1.83)	1.00 (0.65 to 1.55)	0.971
3	0.95 (0.62 to 1.45)	0.79 (0.50 to 1.25)	0.312
4	1.01 (0.67 to 1.53)	0.80 (0.50 to 1.26)	0.329
5 (least)	1.22 (0.84 to 1.77)	1.01 (0.67 to 1.53)	0.960
**ASA grade**			
1 (healthy)	0.07 (0.02 to 0.28)	0.13 (0.03 to 0.57)	0.007
2	0.30 (0.23 to 0.39)	0.37 (0.28 to 0.50)	< 0.001
3	Reference	Reference	
4	1.62 (1.04 to 2.53)	1.60 (1.20 to 2.58)	0.052
5 (moribund)	12.91 (1.66 to 100.51)	11.38 (1.42 to 91.19)	0.022
**Delirium status:** 4AT ≥ 4	4.09 (2.95 to 5.68)	2.80 (1.97 to 3.96)	< 0.001
**COVID-19-positive (< 30days)**	2.79 (1.58 to 4.92)	2.85 (1.54 to 5.28)	< 0.001

ASA, American Society of Anesthesiologists; CI, confidence interval; OR, odds ratio; SIMD, Scottish Index of Multiple Deprivation.

**Table V.** Uni- and multivariable logistic regression analysis of factors associated with readmission to an acute hospital from home within 180 days. Multivariable analysis adjusted for age, sex, Scottish Index of Multiple Deprivation, American Society of Anesthesiologists grade, and COVID-19 within 30 days.

Variable	OR (95% CI)		p-value
	Univariable analysis	Multivariable analysis	
**Age**			
Per year	1.04 (1.03 to 1.06)	1.03 (1.02 to 1.05)	< 0.001
**Sex**			
Male	Ref	Ref	
Female	0.76 (0.55 to 1.04)	0.74 (0.53 to 1.04)	0.081
**SIMD quintile**			
1 (most deprived)	Ref	Ref	
2	0.74 (0.45 to 1.21)	0.69 (0.41 to 1.17)	0.166
3	0.66 (0.39 to 1.10)	0.59 (0.34 to 1.03)	0.063
4	0.86 (0.52 to 1.43)	0.79 (0.46 to 1.37)	0.408
5 (least)	0.83 (0.52 to 1.32)	0.81 (0.49 to 1.34)	0.409
**ASA grade** *			
1 (healthy)	0.07 (0.02 to 0.30)	0.11 (0.03 to 0.47)	0.003
2	0.41 (0.30 to 0.57)	0.48 (0.35 to 0.67)	< 0.001
3	Ref	Ref	
4	1.88 (0.94 to 3.75)	1.96 (0.95 to 4.02)	0.067
5 (moribund)	-	-	-
**Delirium status**: 4AT ≥ 4	2.56 (1.50 to 4.35)	1.79 (1.02 to 3.15)	0.041
**COVID-19-positive (< 30days)**	2.9 (1.13 to 7.45)	2.72 (1.02 to 7.25)	0.040

* Patients discharged out of catchment area were excluded from readmission analysis. No patients with ASA grade 5 went home.

ASA, American Society of Anesthesiologists; CI, confidence interval; OR, odds ratio; SIMD, Scottish Index of Multiple Deprivation.

### Supplementary analyses

Demographics of the 394 patients admitted from higher care settings are provided in Supplementary Table i, and outcomes for these patients in Supplementary Table ii. The mortality rate in these patients was 37.8% within 180 days, and 65% were discharged to a care home. In multivariable analysis adjusting for age, sex, SIMD, ASA grade, COVID-19 within 30 days, and type of residence, delirium was independently associated with an increased mortality rate within 180 days (Supplementary Table iii). Admission from a higher care setting was associated with a two-fold increased mortality rate within 30 days, and nearly two-fold greater mortality rate within 180 days.

### Sensitivity analyses

Of the 1,734 patients with a 4AT score admitted during the study period, 1,185 (68.3%) had an admission 4AT score recorded in the ED. Of these, 272 patients (23.0%) had delirium. Of 1,340 patients admitted directly from home, 975 (72.8%) had an initial 4AT score recorded in the ED. Of these, 119 (12.2%) had delirium. In multivariable analyses adjusting for age, sex, SIMD, ASA grade, and COVID-19 within 30 days, associations of delirium on admission with a higher mortality rate at 180 days, increased likelihood of requiring post-acute inpatient rehabilitation, and readmission within 180 days remained significant (Supplementary Tables iv to vi).

## Discussion

This study reported the prevalence of delirium and associated outcomes in patients admitted directly from home with an acute hip fracture, using robust interrogation of standardized, validated routine data. Only 4.8% of patients did not have a 4AT score recorded in their EHR. More than one in seven patients admitted from home had delirium. After adjusting for age, sex, level of social deprivation, ASA grade, and COVID-19 infection within 30 days, delirium was independently associated with an increased mortality rate at 180 days, nearly three-fold increased risk of requiring post-acute rehabilitation following the acute stay, and nearly two-fold increased readmission risk within 180 days. Observed associations persisted in sensitivity analyses, including only patients with a 4AT score recorded in the ED. This study adds to the existing literature, by demonstrating that delirium can feasibly be ascertained at scale in routine practice in a large, near-complete acute hip fracture population, and that clinically ascertained delirium is associated with unfavourable outcomes in these patients.

Few studies have assessed prevalence and outcomes of delirium in patients admitted directly from home, who likely have lower levels of frailty, dementia, and comorbidities associated both with delirium and adverse outcomes, and in whom days at home following admission with a hip fracture has been demonstrated to be a valid and important patient-centred outcome.^21,31^ The reported prevalence of delirium (14.1%) in this study is slightly lower than reported by Krogseth et al^8^ (19%). This may reflect the much smaller, selected sample with high levels of missing data in the latter study (85/207 did not have a preoperative delirium assessment), and different assessment methods (4AT embedded in routine practice, versus Confusion Assessment Method-based specialist assessment). In this study, prevalence of delirium in the whole cohort (26.5%; 23.0% in those with 4AT scores in ED) is similar to reported prevalences of POD in other hip fracture cohorts, including studies using NHFD data.^1,16-18^

Our observed associations of delirium with adverse outcomes are aligned with those reported in a USA-based study using data from the NSQIP, in which preoperative delirium was ascertained retrospectively using ICD-9 codes and outcomes were assessed up to 30 days.^32^ Our findings highlight the importance of systematic delirium assessment on admission in this vulnerable population, suggesting this could guide early prognostication, facilitate communication with patients and relatives, improve resource allocation, and ensure delivery of specialized multidisciplinary services associated with improved outcomes.^33,34^ Tools such as the 4AT are quick to use and can be widely implemented by non-specialist clinicians.

There was no significant difference in the prevalence of delirium in patients with COVID-19 infection versus those without, despite delirium being a well-documented symptom of COVID-19 during the time period of this study.^35,36^ This may reflect the many other precipitating factors for delirium in patients with an acute hip fracture, and/or the relatively small number of patients admitted from home with COVID-19 within 30 days (n = 53). COVID-19 was independently associated with a higher 30-day mortality and need for post-acute inpatient rehabilitation, consistent with the findings from a previous study using the IMPACT dataset.^23^ Higher rates of delirium were observed in older patients and those with higher ASA grade, consistent with previous studies.^37,38^ The effect size of delirium was reduced by inclusion of ASA grade in multivariable models, suggesting that observed outcomes of delirium may partly reflect its associations with comorbidity. However, effects of delirium remained significant, suggesting either an independent effect of delirium, or the presence of unmeasured factors. Notably, frailty status and dementia were not measured or controlled for, both of which are associated with increased risk of delirium, and with adverse outcomes.^31^ ASA grade may partly control for frailty; in a study of postoperative outcomes in patients undergoing unscheduled surgery, associations between the Clinical Frailty Scale (CFS) score and unfavourable outcomes were not independent of ASA grade.^39^ Our study highlights the unaddressed need for robust prospective frailty assessment in routine hip fracture care, to better stratify and communicate risks. Cognitive impairment has been described as the primary risk factor for institutionalization following hip fracture.^40^ Linkage of EHR data with other routine datasets could facilitate identification of these and other confounding variables.

This study has several strengths. Delirium was prospectively ascertained, with assessment performed during routine care rather than for research purposes. This supports generalizability. Assessment was performed on admission, using a well-established delirium assessment tool with high sensitivity and specificity for delirium.^26^ This study used an established mechanism for data collection and verification by trained specialist auditors, which has been used in peer-reviewed studies published by the IMPACT Collaborative and SHFA, as well as in annual reporting by Public Health Scotland endorsed by the Scottish Government.^29,30^ Hip fracture services at this centre are standardized and delivered according to the Scottish Standards of Care for Hip Fracture Patients (SSCHFP).^3^ This has led to collection of granular data with high levels of delirium ascertainment. The current study employed an additional data validation process conducted by a study author (AJH), independent of the original auditors. A 180-day follow-up period facilitated assessment of outcomes in a group of patients from home with lower risk of adverse short-term outcomes.

A limitation was that dementia and frailty were not measured and adjusted for. Although a recent study demonstrated that EHRs can be used by non-orthogeriatricians to assign an accurate retrospective frailty score,^41^ this was not performed due to large patient numbers. Similarly, estimation of dementia prevalence for the whole cohort, for example through accessing GP records, was not performed due to resource constraints. Nevertheless, delirium, as ascertained by a robust assessment tool embedded in routine clinical practice and EHR, provided valuable prognostic information. Although the vast majority of 4ATs were performed at or near admission as per local clinical and national SSCHFP protocols, precise timing of the first 4AT score in relation to the time of admission was not recorded. However, sensitivity analyses were performed including only 4AT scores from the ED (all within a few hours of admission), and key associations remained. This study was conducted in a single centre, which may introduce bias with regard to the study population and clinical management factors. However, the sample is consecutive, relatively large, and the centre delivers care in accordance with the SSCHFP, with performance consistent with other Scottish centres. Data on periprosthetic fractures were not collected for national audit purposes at the time of the study; this may limit generalisability to these patients. Cohort studies involving retrospective data analysis are vulnerable to bias during data aggregation; risk was minimized by two-stage independent validation of the clinical audit data.

In summary, one in seven patients admitted from their own home had delirium on admission assessment. Adjusting for age, sex, ASA grade, level of social deprivation, and COVID-19 infection, delirium in these patients was independently associated with an increased mortality rate at 180 days, increased risk of requiring post-acute rehabilitation, and increased likelihood of being readmitted to acute services within 180 days of discharge home. Assessment for delirium on admission with a tool such as the 4AT should be part of routine care for hip fracture patients. This could guide early prognostication, facilitate proactive onward care planning, and identify modifiable clinical risk factors. Future studies should seek to identify mediators of adverse outcomes for patients admitted with delirium, and link data from the acute admission with other datasets to better characterize risk factors for delirium.

**Take home message**

- Delirium can be ascertained in a large, near-complete hip fracture population in routine clinical practice using a well-validated, easy-to-use tool (4AT).

- One in seven patients admitted from their own home had delirium, and this was associated with adverse outcomes.

- Delirium assessment on admission using a tool such as the 4AT should be part of standard care for all acute hip fracture patients.

- Early delirium assessment could guide prognostication, facilitate proactive onward care planning, and be used to identify modifiable clinical risk factors.
